# Green Vision in a Tabetic Patient

**Published:** 1899-03-25

**Authors:** 


### GREEN VISION IN A TABETIC PATIENT.

At the last meeting of the Ophthalmological Society Mr. Work Dodd related the case of a patient, aged 32 years, suffering from tabes dorsalis, who was the subject of green vision. In 1886 his sight was quite good; in 1891 he had diplopia, from which he recovered. His colour vision remained good until 1897, and he complained of defective sight shortly before being seen in July, 1898. When first seen the vision of the right eye was $\frac{2}{60}$, and that of the left eye $\frac{1}{60}$. He had Argyle-Robertson pupils, optic atrophy, and contraction of his visual fields; his gait was ataxic, and he had occasional trouble in passing urine. In September, 1898, the vision of the right eye was reduced to $\frac{1}{60}$, while with the left eye he could only perceive movements of the hands. He then saw everything a bright emerald green; it appeared to him as if there was a green veil hung before his eyes, through which he saw everything. He occasionally saw rose-pink spots through the green veil in places. The colours increased in intensity when he was tired, especially the rose-pink. He also had occasional sensations of very brilliant light.

